# Minimum Electromyography Sensor Set Needed to Identify Age-Related Impairments in the Neuromuscular Control of Walking Using the Dynamic Motor Control Index

**DOI:** 10.3390/s24237442

**Published:** 2024-11-21

**Authors:** Ashley N. Collimore, Ryan T. Pohlig, Louis N. Awad

**Affiliations:** 1 Department of Physical Therapy, Sargent College of Health and Rehabilitation Sciences, Boston University, Boston, MA 02215, USA; acollimo@bu.edu; 2 Biostatistics Core Facility, University of Delaware, Newark, DE 19713, USA; rpohlig@udel.edu

**Keywords:** aging, gait, muscle activity, electromyography, motor control, walking

## Abstract

The dynamic motor control index is an emerging biomarker of age-related neuromuscular impairment. To date, it has been computed by quantifying the co-activity of eleven lower limb muscles. Because clinics that routinely employ electromyography typically collect from fewer muscles, a reduced muscle sensor set may improve the clinical usability of this metric of motor control. This study aimed to test if commonly used eight- and five-muscle electromyography (EMG) sensor sets produce similar dynamic motor control indices as the previously examined eleven-muscle sensor set and similarly differentiate across age subgroups. EMG data were collected during treadmill walking from 36 adults separated into young (N = 18, <35 yrs.), young-old (N = 13, 65–74 yrs.), and old-old (N = 5, ≥75 yrs.) subgroups. Dynamic motor control indices generated using the sensor set with eleven muscles correlated with the eight-muscle set (R^2^ = 0.70) but not the five-muscle set (R^2^ = 0.30). Regression models using the eleven-muscle (χ^2^(4) = 10.62, *p* = 0.031, Nagelkerke R^2^ = 0.297) and eight-muscle (χ^2^(4) = 9.418, *p* = 0.051, Nagelkerke R^2^ = 0.267) sets were significant and approaching significance, respectively, whereas the model for the five-muscle set was not significant (*p* = 0.663, Nagelkerke R^2^ = 0.073). In both the eleven-muscle (Wald χ^2^ = 5.16, *p* = 0.023, OR = 1.26) and eight-muscle models (Wald χ^2^ = 4.20, *p* = 0.04, OR = 1.19), a higher index significantly predicted being in the young group compared to the old-old group. Age-related differences in the neuromuscular control of walking can be detected using dynamic motor control indices generated using eleven- and eight-muscle sensor sets, increasing clinical usability of the dynamic motor control index.

## 1. Introduction

Adults aged 65 and older are one of the fastest-growing populations [1]. With increasing age, physiological and neuromotor changes, such as muscle atrophy, reduced central neural drive [2], and impaired muscle coordination [3,4], lead to mobility impairments, including poor balance [5,6,7], slower walking speeds [5,7,8,9], and reduced muscle strength [6,9,10,11,12,13]. Mobility impairments, in turn, result in a more sedentary lifestyle, with reduced community participation [14] and an increase in fall risk [6,9,14] compared to younger adults. Prognostic biomarkers of mobility impairment that can be incorporated into standardized clinical assessments of mobility [15,16] have the potential to identify age-related functional decline *before* it happens, enabling early, targeted interventions that can reduce long-term mobility deficits and promote healthier aging. 

Among older adults, changes in neuromuscular function (i.e., impaired muscle recruitment and coordination) may occur before functional mobility begins to decline [15,16]. While there are myriad approaches to measuring neuromuscular function [2,4,15,16,17,18,19], approaches based on evaluating muscular co-activation during functional activities have produced two promising measurements of impaired neuromuscular function. The first, known as muscle synergies, is defined as the number of different groups of muscles co-activated in time with task demands [20,21,22,23]. The number of muscle synergies observed during a task has been widely used to describe impairments in neuromuscular function [4,19,23,24], and a reduced number of muscle synergies during walking is associated with increased fall risk [25,26]. Though popular, standard muscle synergy analysis techniques are highly impacted by the data filtering and processing methods chosen, which have not been standardized. Different pre-processing decisions are known to impact the number of synergies observed in a dataset, making the approach an inconsistent indicator of impaired neuromuscular function [21,27,28,29]. Moreover, the discrete nature of the number of muscle synergies limits its ability to differentiate across clinical presentations (i.e., individuals can have the same number of muscle synergies but different levels of mobility impairment). Indeed, the muscle synergy approach has not shown impaired neuromuscular function in older adults [30,31,32] despite their known walking impairments [5,7,8,9], suggesting it may not be a sensitive metric for identifying age-related impairments in neuromuscular function, especially at the early time points required to initiate timely intervention.

In prior research, we presented an alternative measure of age-related neuromuscular impairment [29]: the dynamic motor control index. The dynamic motor control index was shown to effectively identify age-related differences in neuromuscular function, whereas the number of muscle synergies could not, suggesting it may be a more meaningful biomarker for age-related neuromuscular impairment. Whereas muscle synergy analyses employ factorization techniques to identify the number of synergies that explain the observed movement, the dynamic motor control index seeks to identify the total variability accounted for (VAF) by the one-muscle synergy solution. The VAF is then scaled to a z-score, centered around 100, based on a reference group [24]; the resulting index value quantifies muscular co-activation relative to the reference group, with lower values thought to be indicative of the degree of neuromuscular impairment. Proper selection of the reference group is thus critical to successfully use the dynamic motor control index for identifying impaired neuromuscular function. 

Steele et al. originally developed the dynamic motor control index as a clinical tool that was both easy to implement and easy to interpret; by scaling the metric around the average of a salient reference group, clinicians can ascertain if and how much a participant is below the average score of 100 [24]. In our prior study of the dynamic motor control indices of older adults, we proposed that the dynamic motor control index could be incorporated into regular clinical evaluations to facilitate the timely detection of age-related functional decline. However, the long setup time required for electromyography (EMG) data collection is a well-documented barrier to the clinical usability of these metrics [33,34]. In our prior work, we utilized an eleven-muscle set under the premise that more muscles provide a deeper understanding of neuromuscular function, but requiring eleven muscles may also hinder clinical usability [33,34]. Clinical settings that typically employ EMG measurement approaches use between four and eight muscles [24,29,35,36,37], and eight- and five-muscle sets have previously been used in clinical settings that use the dynamic motor control index in evaluations of children with cerebral palsy [24,37].

Reducing the muscle set needed to compute a meaningful dynamic motor control index would improve the potential clinical usability of the dynamic motor control index for identifying age-related impairments in neuromuscular control. However, as a measure of muscle co-activity, the composition of the muscle set used may impact its measurement abilities [38]. Though others have used eight- and five-muscle sets to measure differences in neuromuscular control between those with versus without neurological injury, there is a gap in the field’s knowledge of whether these same muscle sets can identify the neuromuscular differences due to aging, which results from different mechanisms. The aim of this study was to test the hypothesis that the commonly used eight- and five-muscle EMG sensor sets would produce similar dynamic motor control indices in older adults as our previously examined eleven-muscle set and similarly differentiate across age subgroups.

## 2. Materials and Methods

### 2.1. Participants

As described in our prior work, EMG data from 38 adults between 18 and 80 years old and with no known neurological impairments were gathered from a publicly available dataset [32], with subjects grouped into young (<35 years old; YA), young-old (65–74 years old; YO), and old-old (≥75 years old; OO) subgroups [39] based on prior work demonstrating between-group differences in the amount of sedentary time [40], quality of life [40], and prevalence of frailty and chronic conditions [41,42]. All procedures were approved by the Ethics Committees of the Humboldt-Universität zu Berlin, Kassel University, and Heidelberg University.

### 2.2. Data Collection and Processing

All data collection procedures have been previously described by Santuz et al. [32]. Briefly, EMG data were collected from the right lower limb muscles during comfortable treadmill walking. For our analyses, we re-analyzed the raw EMG data from 1 min of treadmill walking (i.e., the second unperturbed walking trial from experimental protocol E3) with the young cohort walking at 1.2 m/s and young-old and old-old cohorts walking at 1.1 m/s based on average comfortable speed in a pilot study [32].

**Figure 1:**
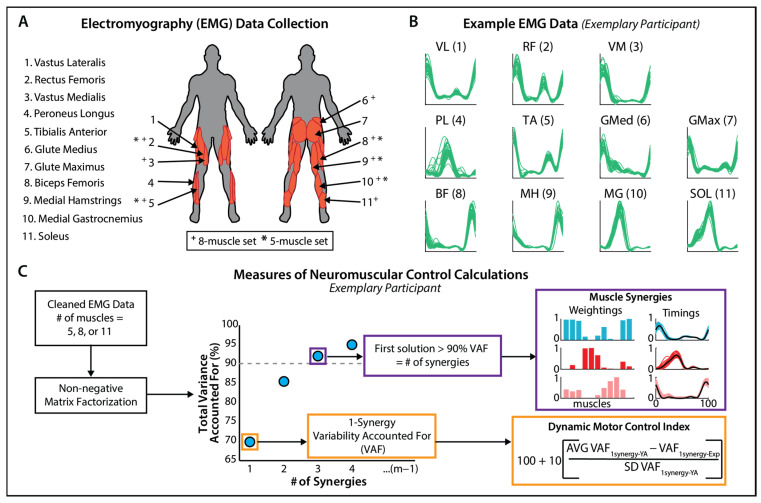
(**A**) EMG data were collected from 11 different muscles. Reduced muscle sets of 8 and 5 muscles were selected for continuity with prior research; (**B**) Exemplary EMG data from the 11 different muscles for one participant; (**C**) Dynamic motor control index and muscle synergy calculations for an exemplary participant. EMG data from 5, 8, or 11 muscles were inputted into a non-negative matrix factorization algorithm. This algorithm calculates the best solution for 1 synergy up to (#muscles—1) synergies and reports the solution’s variability accounted for (VAF). The 1-synergy solution is used to calculate the dynamic motor control index. The first solution with >90% of the VAF (or until a new synergy does not increase VAF by at least 5%) is the muscle synergy solution.

Eleven right lower limb muscles: vastus medialis, rectus femoris, vastus lateralis, soleus, medial gastrocnemius, peroneus longus, tibialis anterior, biceps femoris, medial hamstrings, gluteus maximus, and gluteus medius were used to calculate the dynamic motor control index values and the number of muscle synergies. Subsets of eight muscles [37] and five muscles [24,29] from this dataset were created to calculate the new dynamic motor control index values (Figure 1A); the muscle composition for the eight- and five-muscle sets matched the prior work that used the dynamic motor control index to successfully differentiate between those with and without neurological injury. The muscles removed in these subsets were not the sole dominant contributor to their respective synergies, suggesting they are redundant with the remaining muscles and that impaired neuromuscular function can still be captured in their absence [38].

As previously described, custom MATLAB (MathWorks, Natick, MA, USA) scripts were used to clean EMG signals (Figure 1B), resulting in 30 strides for subsequent analyses [39].

Non-negative matrix factorization using a modified version of a publicly available MATLAB script [43] was used to calculate the number of muscle synergies and the dynamic motor control indices. Non-negative matrix factorization is a mathematical technique used to divide a matrix of data into two vectors that explain most of the variability in the data; in this case, the vectors represent muscle composition, meaning which muscles are co-activated, and the timing, which is when in the gait cycle the synergy is in use. This algorithm calculates the best solution for a given number of synergies, in this case from 1 synergy to the (#muscles—1) synergies and reports the solution’s variability accounted for (VAF). Non-negative matrix factorization data is often used for electromyography data to ensure the resulting vectors are never negative, as muscle activity cannot be negative [44]. The number of muscle synergies was defined as the minimum number needed to achieve 90% VAF or until the addition of a new synergy did not increase the total VAF by more than 5%. The dynamic motor control index was calculated using the VAF of the one-muscle synergy solution to the non-negative matrix factorization and then converted into the dynamic motor control index using the following equation [24] (Figure 1C):

(1) $$\mathrm{Dynamic}\;\mathrm{Motor}\;\mathrm{Control}\;\mathrm{Index}=\;100+10(\frac{{AVG\;VAF}_{1\;synergy-Control\;}-{VAF}_{1\;synergy-Exp}}{\;{SD\;VAF}_{1\;synergy-Control}})$$

As previously defined, the *AVG VAF_1synergy−Control_* is the average of the VAF of the one-synergy solution of the young adult control group, *SD VAF_1synergy−Control_* is the standard deviation of the VAF of the one-synergy solution of the young adult control group, and *VAF_1synergy−Exp_* is the VAF of the one-synergy solution for each individual in the experimental groups (i.e., young-old and old-old adults) [39]. The index is centered around a value of 100, and each 10-point change represents a dynamic motor control index that is one standard deviation from the control group. The process of non-negative matrix factorization, calculation of the number of muscle synergies, and conversion of the one-muscle synergy VAF into a dynamic motor control index was completed for each participant for each of the three muscle sets.

### 2.3. Data Analysis

Bivariate correlations were used to compare dynamic motor control indices calculated from the eleven-muscle set and the eight- and five-muscle sets. Multinomial logistic regression models were then used to test whether the reduced muscle sets would be able to differentiate among age-related subgroups. These models used the old-old subgroup as the reference comparison for the younger and young-old subgroups. As in our prior work, the number of muscle synergies for each participant was added to each model as a covariate (Figure 1C). The analysis was run in two blocks; the first contained the main effects of the dynamic motor control index and the number of muscle synergies, and the second included the interaction between the two variables. The dynamic motor control index and the number of muscle synergies were both mean-centered. We sought to determine if the dynamic motor control index remained a significant predictor (*p* < 0.05) of age-subgroup in the reduced muscle set models.

## 3. Results

**Figure 2:**
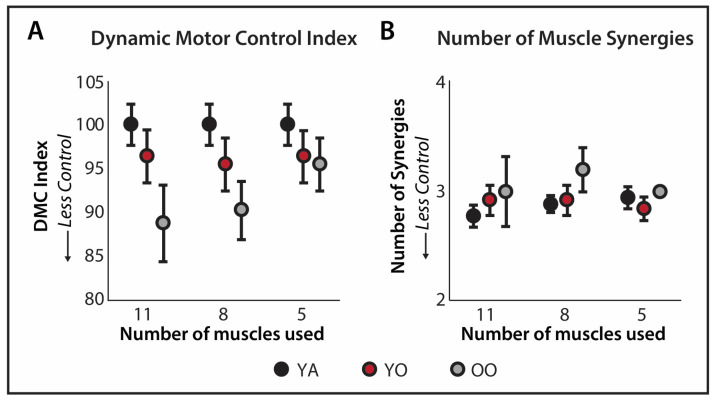
(**A**) Average ± standard error dynamic motor control indices and (**B**) number of muscle synergies computed for the 11, 8, and 5 muscle sensor sets.

Two individuals were excluded from the analysis for reasons previously described [39], resulting in 36 total participants in the analysis. On average, the young subgroup (N = 18 participants; 11 female) was 27 ± 3 years old, the young-old subgroup (N = 13 participants; 10 female) was 70 ± 3 years old, and the old-old subgroup (N = 5 participants; 3 female) was 78 ± 2 years old. By definition, the dynamic motor control index for the young group was 100 ± 10 for all three muscle sets. For the young-old group, the dynamic motor control indices were 96.4 ± 10.79 for the eleven-muscle set, 95.49 ± 10.82 for the eight-muscle set, and 96.40 ± 10.69 for the five-muscle set. For the old-old group, the dynamic motor control indices were 88.86 ± 9.67, 90.35 ± 7.31, and 95.52 ± 6.74 for the eleven-, eight-, and five-muscle sets, respectively (Figure 2). The number of muscle synergies for each muscle set for each subgroup is reported in Table A1.

**Figure 3:**
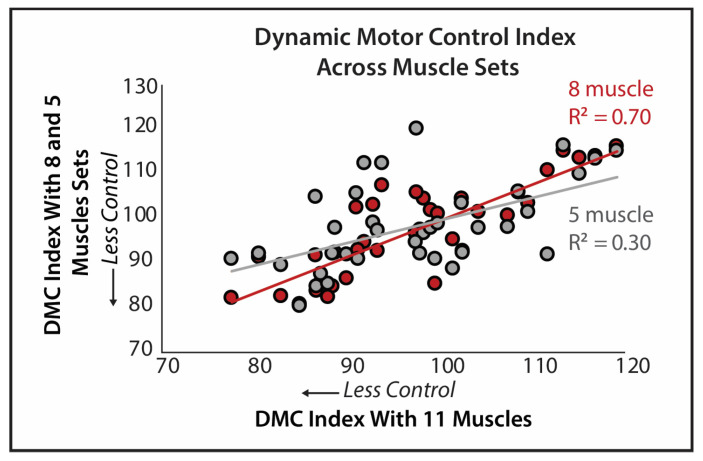
Dynamic motor control indices of the 8-muscle set are highly correlated with the 11-muscle set (R^2^ = 0.70), while the 5-muscle set is not (R^2^ = 0.30).

Dynamic motor control indices generated using the eleven-muscle set were highly correlated with the indices generated using the eight-muscle set (R^2^ = 0.697, *p* < 0.001) but only moderately correlated with the indices generated using the five-muscle set (R^2^ = 0.298, *p* < 0.001; Figure 3). Similarly, the multinomial regression models predicting young, young-old, and old-old subgroups by the main effects of the dynamic motor control index and the number of muscle synergies were significant and approaching significance, respectively, for the indices generated using the eleven-muscle (χ^2^(4) =10.62, *p* = 0.031, Nagelkerke R^2^ = 0.297; Table 1) and eight-muscle (χ^2^(4) = 9.418, *p* = 0.051, Nagelkerke R^2^ = 0.267; Table 1) muscle sets, whereas the model for the five-muscle set was not significant (*p* = 0.663, Nagelkerke R^2^ = 0.073; Table 1). A higher dynamic motor control index was significantly predictive of being in the young group compared to the old-old group after adjusting for the number of muscle synergies in both the eleven-muscle (Wald χ^2^ = 5.16, *p* = 0.023, OR = 1.26) and eight-muscle set models (Wald χ^2^ = 4.20, *p* = 0.04, OR = 1.19). Additionally, in the eight-muscle set model, a lower number of muscle synergies (Wald χ^2^ = 3.911, *p* = 0.048, OR = 0.027) was significantly predictive of being in the young group compared to the old-old group. When the interaction between the dynamic motor control index and the number of muscle synergies was tested, it was not significant in the eleven-muscle set (*p* = 0.06) or the five-muscle set (*p* = 0.126) models. The model could not converge with the inclusion of the interaction in the eight-muscle set model.

## 4. Discussion

Electromyography (EMG) is a powerful assessment tool with the potential to identify age-related neuromuscular changes that may precede functional decline. Our previous study demonstrating that the dynamic motor control index could measure age-related deficits in the neuromotor control of walking suggested that incorporating evaluations of the dynamic motor control index into periodic clinical evaluations could assist with early identification of age-related impairments in walking function. The findings of this study extend this work and suggest that a standard set of eight lower limb EMG sensors may be the minimum needed to compute dynamic motor control indices that can identify age-related differences in neuromuscular function. The subset of eight muscles chosen for this study, which excluded the vastus lateralis, peroneus longus, and gluteus maximus, was able to both produce similar dynamic motor control indices to the eleven-muscle set that we previously validated, as well as similarly differentiate between the age subgroups. This is an important finding that supports the clinical usability of this metric of motor control; the overall number of muscles needed for identifying age-related impairments with the dynamic motor control index can be reduced from eleven to eight, alleviating some testing burden while ensuring the validity of the metric with a different composition of muscles.

Contrary to our hypothesis, the commonly used five-muscle set was unable to differentiate between young, young-old, or old-old subgroups. Consequently, at this time, we cannot recommend that clinicians use a five-muscle set to identify age-related impairments in neuromotor control using the dynamic motor control index. This finding deviates from prior work in children with and without cerebral palsy, in which the dynamic motor control index calculated with the same five-muscle set was significantly different between neurotypical healthy controls and all levels of impairment, as well as between different impairment levels [24]. The five-muscle set may not have been successful in our analysis of age-related neuromuscular decline for two potential reasons. The first is that simple treadmill walking may not have been difficult enough to elucidate impairments in neuromuscular function. Prior work in older adults has been more successful in identifying neuromuscular impairments during fast walking [45], walking on uneven surfaces [46], or taking a step [31]. Even overground walking may reveal more neuromuscular impairments than treadmill walking [47,48]. Indeed, the aforementioned work in cerebral palsy collected data during overground trials, while this study’s older adult data were collected on the treadmill. Thus, differences in the task may be contributing to the differences in results seen across the two populations.

Second, the soleus and gluteus medius, two of the muscles not used in the five-muscle set examined in this study, may ultimately be important for identifying changes in neuromuscular function due to aging. Several studies have shown neuromuscular changes in the soleus of older adults compared to younger adults, including reduced excitability [49], reduced H-reflex with longer latency [50,51], and increased co-contraction with the tibialis anterior [52]. Similarly, when compared to younger adults, in older adults, the gluteus medius has been shown to develop lower forces and contribute less to support during walking [53] and to increase activity in sit-to-stand activities [54], obstructed walking [55], and perturbed walking [56]. Moreover, gluteal muscles have a high level of atrophy due to aging, which is associated with falling [57,58]. These results suggest that alternative five-muscle sets that capture both the soleus and gluteus medius may be important for measuring neuromuscular changes due to aging. That is to say, different populations of interest may require different five-muscle sets.

Our findings that a five-muscle set may not perform similarly to an eleven-muscle set are supported by a recent simulation study [38]. When comparing a five-muscle set to a 29-muscle set, the similarity in muscle synergy analysis was only 57%, with the similarity increasing to 80% when over ten muscles were included. Furthermore, the study also showed that the fewer muscle subsets over-estimated VAF, which is used to calculate the dynamic motor control index. Selecting muscles with large isometric force or dominant muscles from the synergy analysis for the reduced muscle sets may produce better results than our study’s investigation of standard muscle sets previously used. Future work should employ this approach for identifying alternative reduced muscle sets.

### Limitations

An interesting finding from the current study was that the number of muscle synergies was not a significant predictor of age group when using the eleven-muscle set but became significant when using the eight-muscle set, with old-old adults having a *higher* number of synergies than young adults. This is not aligned with previous work showing a *reduced* number of synergies with neuromuscular impairment [19,24] and is likely due to the small sample size of five old-old adults in this study. A second limitation of this work is that data were only collected from the right limb. Changes in neuromuscular control due to aging may happen unilaterally, and, in the future, electromyography data from both limbs should be collected and compared to ensure that all possible changes in neuromuscular control are detected. A third limitation is that we focused on comparing the dynamic motor control index to the number of muscle synergies and did not investigate the potential contribution of the muscle composition or timing of individual synergies. These may be complementary measures to the dynamic motor control index as they may be able to characterize where in the gait cycle the neuromuscular deficit is occurring, which the dynamic motor control index cannot do alone. Finally, as this study used an open-source dataset, we did not have access to participant’s lifestyles, walking biomechanics, or other data that may provide additional insight into the neuromuscular control of the participants in the study. While the goal of this study was to use the known general age-related neuromuscular decline as a proxy for evaluating the dynamic motor control index, future work should measure dynamic motor control indices in conjunction with these additional variables to further validate these results.

## 5. Conclusions

The major finding of this work is that age-related differences in the neuromuscular control of walking can be detected using dynamic motor control indices generated using the eleven- and eight-muscle EMG sensor sets studied but not the five-muscle EMG sensor set. More specifically, we found that the removal of the gluteus medius, vastus medialis, and soleus EMG sensors from the muscle sensor set resulted in dynamic motor control indices being unable to identify age-related differences in the neural control of walking, suggesting the importance of these muscles in detecting gait impairment. The clinical implication is that incorporating electromyography data from the eight-muscle set into routine assessments of walking function can enable the calculation of dynamic motor control indices capable of aiding in the early detection of age-related impairments. However, because these findings contrast with prior work that successfully used the same five-muscle sensor set to detect neuromuscular differences due to cerebral palsy during walking, testing the performance of the five-muscle sensor set using locomotor tasks that amplify age-related neuromuscular deficits may be worthwhile. Alternatively, different five-muscle sensor sets may be more effective in identifying age-related neuromuscular deficits during comfortable treadmill walking, warranting further investigation.

## Figures and Tables

**Table 1:** Multinomial logistic regression results for eleven-, eight-, and five-muscle sets.

Model Statistics										
		11 Muscles			8 Muscles			5 Muscles		
Model		R²	Χ²	*p*	R²	Χ²	*p*	R²	Χ²	*p*
Muscle Synergies and Dynamic Motor Control Index		0.297	10.620	0.031	0.267	9.418	0.051	0.075	2.396	0.663
Muscle Synergies, Dynamic Motor Control Index, and Interaction		0.422	16.251	0.012	Could not converge			0.193	6.533	0.366
**Predictor Statistics**										
		**11 muscles**			**8 muscles**			**5 muscles**		
**Predictors**		**Χ²**	***p***		**Χ²**	***p***		**Χ²**	***p***	
Constant		11.266	0.004		10.901	0.004		8.189	0.017	
Muscle Synergies		5.416	0.067		5.186	0.075		0.924	0.630	
Dynamic Motor Control Index		9.412	0.009		6.845	0.033		1.525	0.467	
**Subgroup Analysis (OO Reference Group)**										
		**11 muscles**			**8 muscles**			**5 muscles**		
**Predictor**		**β**	**OR**	***p***	**β**	**OR**	***p***	**β**	**OR**	***p***
YH	Constant	2.30		0.025	2.14		0.024	1.38		0.013
	Muscle Synergies	−3.37	0.03	0.019	−3.63	0.03	0.048	−1.23	0.29	0.484
	Dynamic Motor Control Index	0.23	1.26	0.017	0.17	1.19	0.040	0.07	1.07	0.289
YO	Constant	2.05		0.038	1.88		0.050	1.05		0.069
	Muscle Synergies	−2.19	0.11	0.048	−3.00	0.05	0.099	−1.62	0.20	0.369
	Dynamic Motor Control Index	0.17	1.19	0.047	0.12	1.12	0.154	0.04	1.04	0.596
YH	Constant	3.86		0.981	9.18		0.996	1.55		0.807
	Muscle Synergies	−5.84	0.00	0.987	−130.53	0.00	0.997	−5.52	0.00	0.942
	Dynamic Motor Control Index	0.34	1.41	0.056	−0.64	0.527	0.998	−0.11	0.90	0.916
	Muscle Synergies x Dynamic Motor Control Index	−0.36	0.70	0.000	Could not converge			2.12	8.31	0.861
YO	Constant	3.34		0.981	6.98		0.990	2.81		<0.001
	Muscle Synergies	−3.68	0.03	0.987	−98.37	0.00	0.993	−23.77	0.00	.
	Dynamic Motor Control Index	0.27	1.31	0.015	−0.95	0.386	<0.001	0.08	1.09	0.213
	Muscle Synergies x Dynamic Motor Control Index	−0.16	0.86	.	Could not converge			−0.69	0.50	.

## Data Availability

Electromyography data were made publicly available by Santuz et al. (2020). The original contributions presented in the study are included in the article/Supplementary Material. Further inquiries can be directed to the corresponding author/s.

## Supplementary Materials

The following supporting information can be downloaded at https://www.mdpi.com/article/10.3390/s24237442/s1, Data S1: Participant Characteristics and Neuromuscular Complexity.

## Appendix A

**Table A1:** Dynamic Motor Control Index and Muscle Synergies.

	Dynamic Motor Control Indices			Number of Muscle Synergies		
	11 Muscles	8 Muscles	5 Muscles	11 Muscles	8 Muscles	5 Muscles
Young	100 ± 10	100 ± 10	100 ± 10	2.78 ± 0.43	2.89 ± 0.32	2.94 ± 0.42
Young-Old	96.43 ± 10.79	95.49 ± 10.82	96.40 ± 10.69	2.92 ± 0.49	2.92 ± 0.49	2.85 ± 0.38
Old-Old	88.86 ± 9.67	90.35 ± 7.31	95.52 ± 6.74	3.00 ± 0.70	3.20 ± 0.44	3.00 ± 0.00
